# Arterial stiffness and atrial fibrillation recurrence: another risk marker or a call for better management of concomitant disease?

**DOI:** 10.1007/s12471-022-01678-8

**Published:** 2022-03-08

**Authors:** J. R. de Groot, D. Linz

**Affiliations:** 1 Department of Cardiology, Heart Centre, Amsterdam University Medical Centres, University of Amsterdam, Amsterdam, The Netherlands; 2 Department of Cardiology, Cardiovascular Research Institute Maastricht, Faculty of Health, Medicine and Life Sciences, Maastricht University and Maastricht University Medical Centre+, Maastricht, The Netherlands; 3 Department of Cardiology, Radboud University Medical Centre, Nijmegen, The Netherlands; 4 Department of Biomedical Sciences, University of Copenhagen, Copenhagen, Denmark; 5 Centre for Heart Rhythm Disorders, University of Adelaide and Royal Adelaide Hospital, Adelaide, Australia

Atrial fibrillation (AF) is the most common cardiac arrhythmia, and it is associated with increased risk of ischaemic stroke, heart failure and mortality [1]. Cardiovascular risk factors are highly prevalent in patients with AF and vice versa [2]. In the European Society of Cardiology Guidelines for the diagnosis and management of AF, systematic assessment and management of modifiable concomitant cardiovascular risk factors are important pillars of the ABC approach (Anticoagulation/Avoid stroke-Better symptom control-Comorbidities/Cardiovascular risk factor management) [1]. In addition to oral anticoagulation for prevention of stroke, better management of symptoms by heart rate control and restoration of sinus rhythm with antiarrhythmic drugs or through catheter ablation is recommended. Despite enormous progress in ablation technology, interventional treatment of AF with catheter ablation remains a challenge, in particular when the absence of AF during follow-up is considered as an endpoint [3].

Several factors have been identified as predictors of AF recurrence after AF ablation. For example, the APPLE score (attributing one point each to age > 65 years, persistent AF, impaired estimated glomerular filtration rate < 60 ml/minute per 1.73 m^2^, left atrial diameter ≥ 43 mm, left ventricular ejection fraction (LVEF) < 50%) has been introduced as a tool to estimate the risk of AF recurrence after AF ablation. However, aside from the statistically significant predictive value, the accuracy of this score is modest at best [4]. Currently established risk factors for AF recurrence that are commonly used in clinical practice to stratify patients for AF ablation include obesity, left atrial volume index (LAVI), CHA_2_DS_2_-VASc score and presence of persistent or long-standing persistent AF. Additionally, surrogate markers of arterial stiffness, such as pulse pressure, pulse wave velocity and reduced aortic compliance, have been described as independent risk factors for AF [5–7]. Despite evolving evidence supporting the association between AF and arterial stiffness, the association between arterial stiffness and AF recurrence after AF ablation remains unclear.

In this issue of the* Netherlands Heart Journal*, Shchetynska-Marinova and colleagues present their study on the relation of arterial stiffness with AF recurrence after ablation [8]. They report on 151 patients undergoing AF ablation from June 2015 through December 2017 (mean ± standard deviation age 72 ± 10 years, 64% male, 39% persistent AF) in whom arterial stiffness was assessed by measuring aortic distensibility. Mean CHA_2_DS_2_-VASc score was 3.0 ± 1.7, indicating this cohort of patients were not only relatively old—compared with the Dutch situation—but they were also more severely burdened by cardiovascular comorbidities. As a comparison, a recent analysis of the Netherlands Heart Registration database showed that in the Netherlands, the mean age of patients undergoing AF ablation is 60 ± 10 years and the mean CHA_2_DS_2_-VASc score is 1.5 ± 1.3 [9].

In the study by Shchetynska-Marinova et al., hypertension was present in 78% of the patients, heart failure in 43%, chronic kidney disease in 32%, coronary artery disease in 29% and diabetes mellitus in 13% [8]. The authors calculated aortic distensibility by dividing the difference between systolic and diastolic diameter of the descending aorta (measured by periprocedural transoesophageal echocardiography) by the diastolic diameter and then multiplying that by the pulse pressure (measured by brachial blood pressure) [8]. Aortic distensibility was used as a proxy for arterial stiffness throughout the body. Pulmonary vein isolation was performed with voltage abatement and exit block as procedural endpoints. Patients were followed up for a median duration of 21 months (interquartile range 15–31), with routine visits at the outpatient clinic and 72-hour Holter monitoring once (three months after the procedure).

During follow-up, 62.3% of the patients experienced AF recurrence [8]. These patients were older, had a higher CHA_2_DS_2_-VASc score, a higher APPLE score, a higher symptom burden (as assessed by European Heart Rhythm Association score), more chronic kidney disease, a lower LVEF and a larger LAVI and more often took digoxin or amiodarone than those without recurrences. Aortic distensibility was significantly lower in patients with AF recurrence than in those without (1.5 ± 0.7 vs 2.6 ± 2.3 × 10^−3^ mm Hg^−1^, *p* < 0.0001). In multivariable analysis, LAVI (odds ratio (OR) 2.9, 95% confidence interval (CI) 1.2–3.4) and aortic distensibility (OR 3.6, 95% CI 2.8–4.1) remained significant predictors of AF recurrence.

How should we appreciate these findings? The authors make a strong case that aortic distensibility—the proxy they used for arterial stiffness—is an independent predictor of AF recurrence after AF ablation in a cohort of patients heavily burdened by cardiovascular comorbidities. This is true from a statistical point of view, but does it also make sense from a biological perspective?

Aortic distensibility strongly correlated with age, hypertension, kidney failure and heart failure but also with LVEF, LAVI and CHA_2_DS_2_-VASc score. In other words: Does aortic distensibility represent a marker of the underlying cardiovascular disease? In that respect, the finding that aortic distensibility correlated with AF outcome after ablation is not very surprising. How aortic distensibility relates to sex is not reported, but a recent subanalysis of the RACE V (Reappraisal of Atrial Fibrillation: Interaction between HyperCoagulability, Electrical remodelling, and Vascular Destabilisation in the Progression of AF) trial showed that biomarkers of vascular remodelling were predominantly increased in male subjects with paroxysmal AF, whereas females expressed more biomarkers of inflammation [10]. Hence, the role of vascular disease in AF pathophysiology and AF recurrence after ablation may be different for the sexes.

A second consideration is how to appreciate the implication of the predictive value of aortic distensibility for AF recurrence. Is this yet another marker of cardiovascular comorbidity, or does arterial stiffness play a pivotal role in the haemodynamics causing the development and perpetuation of AF, as Shchetynska-Marinova et al. suggest? It remains unclear whether early management of underlying concomitant cardiovascular diseases is effective in preventing the progression of increased aortic distensibility (and decreased arterial stiffness). Another important question is whether and to which extent already existing decreased aortic distensibility (in this study, baseline aortic distensibility was 1.9 ± 1.1 × 10^−3^ mm Hg^−1^) is reversible when potentially modifiable risk factors are managed and controlled.

These questions require further prospective intervention studies and go beyond the current study by Shchetynska-Marinova et al. It remains unclear to which extent the findings in this selected population sample reported by the authors can be extrapolated to a group of consecutive patients referred for AF ablation and how we should appreciate aortic distensibility as a risk marker of AF progression in patients who will not undergo ablation.

In summary, arterial stiffness is associated with the presence of concomitant cardiovascular risk factors and may help to identify AF patients with a higher risk of AF recurrence after ablation. For now, the clear recommendation by current AF management guidelines that cardiovascular comorbidities need to be optimised as part of a holistic approach towards the AF patient remains unchanged. Future intervention studies are needed to determine whether arterial stiffness is a marker of underlying cardiovascular risk factors, which should trigger a comprehensive risk factor management approach, or whether it represents an independent, modifiable risk factor for recurrence of AF in patients undergoing AF ablation [1].
